# Unfolding of Novel Independent Missense Mutations in VAMP2 and AGRN and Their Collective Role in Global Developmental Delay: A Case Report

**DOI:** 10.7759/cureus.28464

**Published:** 2022-08-27

**Authors:** Negar Heidarpour, Adityabikram Singh, Johnna M Caputo, Raquel Barbieri, Vijay S Pampana, Vasudeva G Kamath, Gurjinder Kaur

**Affiliations:** 1 Department of Basic Biomedical Sciences, Touro College of Osteopathic Medicine (TouroCOM), Middletown, USA; 2 Department of Basic Biomedical Sciences, Rutgers New Jersey Medical School, Newark, USA; 3 Department of Neurology, Middletown Medical, Middletown, USA

**Keywords:** congenital myasthenic syndrome (cms), agrin (agrn), global developmental delay (gdd), soluble n-ethylmaleimide-sensitive- factor attachment receptor (snare), vesicle associated membrane protein 2 (vamp2/synaptobrevin2)

## Abstract

Vesicle-associated membrane protein 2 (*VAMP2*) and Agrin (*AGRN*) are crucial proteins in neurotransmission. *VAMP2* is a vesicular protein that facilitates the exocytosis of neurotransmitters. At the same time, *AGRN* plays a critical role in the maintenance and function of neuromuscular junctions. Mutations in the signaling pathway of *VAMP2* and *AGRN* impair proper signaling between the presynaptic and postsynaptic neurons, and can result in neurodevelopmental conditions known as global developmental delay (GDD). This study highlights a presentation of GDD in a patient with concurrent mutations in *VAMP2* and *AGRN*. A three-year-old female child presented with GDD characterized by hypotonia, intellectual disability, and dysphagia. Physical exam exhibited signs of developmental delay and severe muscle weakness. EEG findings were suggestive of a hypsarrhythmia pattern. The ophthalmological evaluation showed partial optic atrophy bilaterally. Therapeutic interventions included Keppra and Topamax, which proved ineffective. The patient’s outcome was inconclusive as care was transferred to another facility. This case study reports the novel appearance of two concurrent mutations: p.Gln76Pro associated with *VAMP2* and p.Gln970Glu associated with *AGRN*. Mutations in *VAMP2* lead to a dysfunctional SNARE complex and inhibit exocytosis of neurotransmitters into the synaptic cleft. Mutations in *AGRN* impair the ability to form and activate postsynaptic nicotinic acetylcholine receptors. Improper signaling between presynaptic and postsynaptic neurons is an important determinant of GDD. We hope that accounting for this mutational pattern will contribute to understanding synapse assembly and help unravel the complex interplay of factors involved in the pathology of neuromuscular disorders and GDD.

## Introduction

Synaptic talk precisely counts on synchronized, well-timed neurotransmitter release initiated by the fusion of synaptic vesicles to presynaptic neuronal endings. Soluble N-ethylmaleimide-sensitive factor attachment protein receptor (SNARE) proteins facilitate membrane fusion and are crucial for the exocytosis of synaptic vesicles [1]. Vesicle-associated membrane protein 2 (*VAMP2*) is a vesicle-bound protein that facilitates in docking of the vesicle on the presynaptic membrane [2]. Active *VAMP2* on the synaptic vesicle (v-SNARE) makes a complex with syntaxin-1 (STX1) and synaptosomal-associated protein and 25kDa (SNAP25) proteins on the presynaptic membrane (target or t-SNARE) called the SNARE complex. Proper formation of the SNARE complex in the postsynaptic membrane facilitates the exocytosis of neurotransmitters into the synaptic cleft [1]. Mutations in the components of the canonical synaptic vesicle fusion SNARE complex (*VAMP2*, STX1, and SNAP25) produce variant phenotypes of autism, intellectual disability, movement disorders, and epilepsy [3]. Specific de novo heterozygous *VAMP2* mutations in distinct individuals present with neurodevelopmental disorder characterized by axial hypotonia, intellectual disability, autistic features along with central visual impairment, hyperkinetic movement disorder, and epilepsy [1].

Another synaptic extracellular matrix, glycoprotein Agrin (*AGRN*), aids in the aggregation of postsynaptic nicotinic acetylcholine receptors (AChR). *AGRN* is secreted from the nerve terminal of spinal motor neurons and binds to low-density lipoprotein receptor-related protein 4 (Lrp4) and muscle-specific receptor tyrosine kinase (MuSK) on the postsynaptic muscle membrane [4]. The agrin-Lrp4-MuSK signaling pathway is primarily involved in developing and maintaining neuromuscular junctions (NMJs) [4]. Congenital myasthenic syndromes (CMSs) include NMJ genetic defects implicating a group of neuromuscular diseases classified by the site of the transmission defect, such as presynaptic, synaptic, and postsynaptic [4]. Missense mutations in AGRN genes have been implicated in CMS8, an autosomal recessive disorder characterized by defects in both pre-and postsynaptic regions [4]. Clinically it presents in childhood with early-onset proximal muscle weakness, easy fatigability, ptosis, extraocular muscle weakness, facial palsy, high-arched palate, respiratory insufficiency, and a narrow thorax [5]. 

Recent studies have reported the individual role of *VAMP2 *variants in impaired neurodevelopment [6] and *AGRN* mutations in causing CMS [7,8]. In this case study, we report the novel occurrence of these two mutations, i.e., a genetic variant of protein *VAMP2* (NM_014232: c.227 A>C [p.Gln76Pro]) and *AGRN* (NM_198576: c.2908 C>G [p.Gln970Glu]) in a young child presenting with global developmental delay (GDD) characterized by hypotonia, intellectual disability, and dysphagia. Mutations that we identified in this case report in the *VAMP2* and *AGRN* pathways possibly impair proper neuronal signaling, affecting both the presynaptic and postsynaptic neuronal levels. We hope that these novel mutational patterns will contribute to understanding the pathology of synapse assembly and neuromuscular disorders and illustrate the complexity of factors that could partake in causing GDD. We sincerely anticipate that this research will raise awareness and improve clinical outcomes for patients with these rare mutations.

## Case presentation

Patient and methods

The patient is a 30-month-old female born at 37 weeks gestational age with a birth weight of 3460 grams, normal head circumference, a pulse of 130 BPM, temperature of 98.2 degrees F, and APGAR scores of 9 and 9 at 1 and 5 minutes. Our patient exhibited signs of developmental delay and severe muscle weakness beginning in infancy. Symptoms began at five months of age and included severe dysphagia, poor eye contact with parents, and the inability to roll over from prone to supine. Furthermore, she experienced brief episodes of blank staring and unresponsiveness. Prenatal history was unremarkable, and there was no family history of developmental delay or mental retardation. The patient was placed on feeding therapy at eight months of age and had otherwise not received any prior clinical workup. 

She presented to Middletown Medical Primary Care in Middletown, New York, at 11 months of age for initial evaluation. On physical exam, she was observed to have a mildly dysmorphic face, low-set ears, and a tented upper lip. The patient was alert but displayed moderate-to-severe hypotonia in all extremities and the trunk. Other findings included poor head control, intellectual disability, minimal-to-absent speech, poor visual fixation, and absence of purposeful hand movements compared to other infants at the same age of 11 months. The patient was given an initial diagnosis of developmental delay and severe hypotonia (Table 1). Recommendations included physical, occupational, and speech/feeding therapy, blood work, genetic testing, MRI of the brain to rule out leukodystrophies and congenital abnormalities, and EEG to rule out an epileptic activity. GI and Ophthalmology specialists were also consulted due to gross feeding and visual abnormalities. 

**Table 1:** Clinical presentation of patient from September 2019 to January 2020. GDD: Global developmental delay.

Dates	Summaries from initial and follow-up visits	Diagnostic testing	Interventions
September 2019	Patient was diagnosed with GDD and moderate-to-severe hypotonia	Recommendations for: -Genetic testing -Routine EEG followed up with 48-hr ambulatory EEG to rule out subclinical seizures -MRI with and without contrast under sedation to rule out leukodystrophies and congenital abnormalities	Referred to medical genetics, recommended for physical therapy, occupational therapy, and speech/feeding therapy
October 2019	Patient presented for EEG testing to rule out seizures	Initial EEG: abnormal frequent generalized spike wave/high-voltage slow wave discharges and disorganized background, suggestive of hypsarrhythmia EEG pattern.	No new interventions were started
October 2019	Patient followed up to discuss results of EEG showing hypsarrhythmia. The parents have endorsed episodes of staring and brief unresponsiveness in the patient but deny any shaking.	Orders written for: -MRI brain with and without contrast -Follow-up EEG, ambulatory 24-hour study before next visit to monitor interictal activity to see any significant improvement after starting Keppra	Keppra solution, 100 mg/mL, 1 mL bid for 1 week, then 2ml bid, orally, every 12 hours for 30 days, referred to Ophthalmology and GI due to swallowing difficulty
December 2019	Patient followed up for repeat EEG to monitor interictal activity after starting Keppra	Repeat EEG: No significant improvement was found in comparison to initial EEG	Recommended to continue Keppra 200 mg twice daily
January 2020	Patient followed up to discuss results of genetic testing	No new diagnostic testing performed	Keppra discontinued due to side effects of drowsiness. Started on Topamax Sprinkle 25 mg, 1 cap twice daily
Care transferred to another facility			

Laboratory blood tests showed an unremarkable complete metabolic panel, total creatine kinase, lactic acid, lactate dehydrogenase, acylcarnitine profile, and homocysteine levels. Testing of urinalysis revealed a normal pattern of organic acids. Genetic testing via polymerase chain reaction (PCR) was negative for fragile X syndrome and sequence analysis, and deletion testing of the mitochondrial genome was negative for any mitochondrial disorders. Initial EEG showed abnormal frequent generalized spike-wave, high-voltage slow wave discharges a disorganized background, suggestive of a hypsarrhythmia EEG pattern. MRI of the brain with and without contrast was unremarkable. The ophthalmological evaluation showed partial optic atrophy bilaterally, indicative of an underlying neurological abnormality. She was started on Keppra 100 mg two times (BID) with a weight of 20 lbs., then 200 mg BID orally every 12 hours. This therapy was discontinued due to the adverse effects of drowsiness, and a repeat EEG done after two months of treatment showed no improvement. She was then started on a trial of Topamax sprinkle capsule 25 mg, one capsule twice daily. Afterward, the patient's care was transferred to another facility for further workup. Unfortunately, informed consent could not be obtained from the patient's kin as the patient's kin never followed up for further appointments.

Genetic analysis

Genetic testing showed that the etiology of the developmental delay was from a novel mutation in the *VAMP2* protein with a nonconservative heterozygous missense mutation, NM_014232: c.227 A>C (p.Gln76Pro), in exon 3 in the *VAMP2* gene on chromosome 17. Genetic testing also showed that the patient had a mutation on chromosome 1 for the *AGRN* gene with the nonconservative heterozygous missense mutation, NM_198576: c.2908 C>G (p.Gln970Glu).

## Discussion

**Figure 1:**
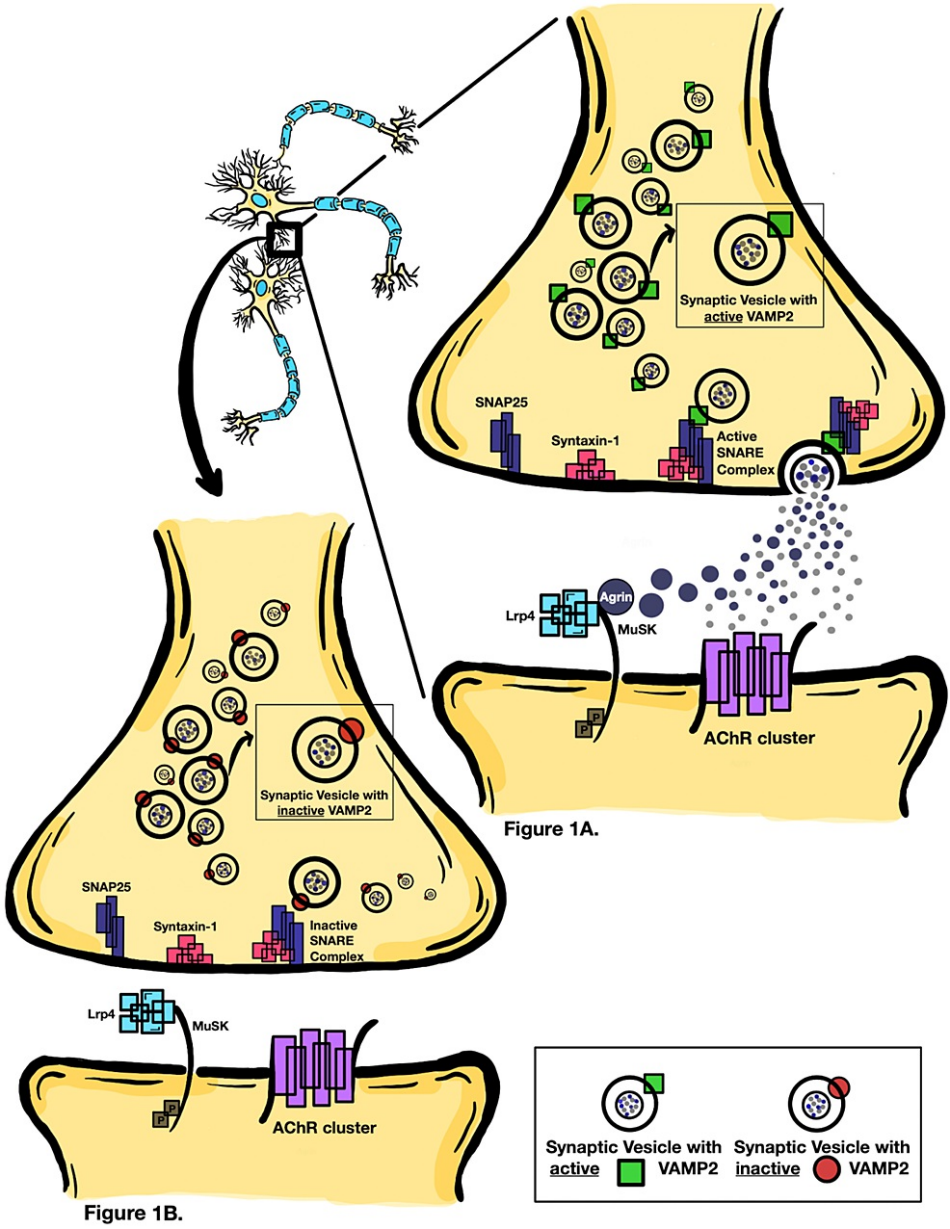
Schematic illustration of VAMP2 protein and AGRN ligand protein signaling pathway and neurotransmitter release. A. Active *VAMP2* on the synaptic vesicle (v-SNARE) makes a complex with syntaxin-1 and SNAP25 on the plasma membrane (target or t-SNARE) for exocytosis of neurotransmitters into the synaptic cleft. Agrin ligand is secreted from the nerve terminal of spinal motor neuron and binds to low-density lipoprotein receptor-related protein 4 (Lrp4; encoded by LRP4) and muscle-specific receptor tyrosine kinase (MuSK; encoded by MUSK) on the postsynaptic muscle membrane. B. Inactive *VAMP2* gene inhibits synaptic vesicles (v-SNARE) from fusing with syntaxin-1 and SNAP25 on the plasma membrane (target or t-SNARE). This will inhibit the docking of the vesicle and not allow exocytosis of neurotransmitters and *AGRN* ligand protein into the synaptic cleft [4,9]. VAMP2: Vesicle-associated membrane protein 2; AGRN: Agrin.

**Figure 2:**
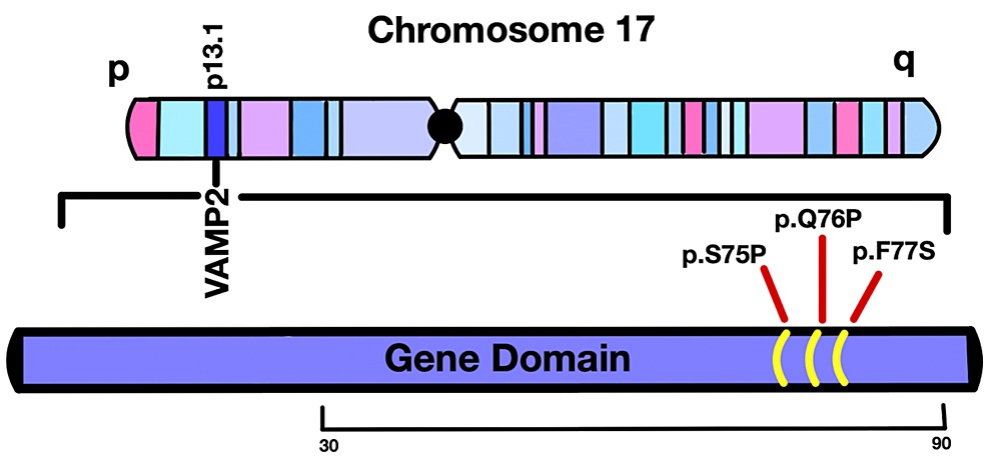
Hypothetical representation of VAMP2 protein in chromosome 17. The *VAMP2* protein in chromosome 17 position 13.1 (GC17M009187) indicates the variants identified in our patient (p.Q76P) in relation to patient variants found in Salpietro V et al. (p.S75P and p.F77S) [1].

Earlier studies reported that mutations in the components of the canonical synaptic vesicle fusion SNARE complex (*VAMP2*, STX1, and SNAP25) inhibit docking of the vesicle preventing the exocytosis of neurotransmitters and release of* AGRN* ligand protein into the synaptic cleft [3,4] (Figures 1A-1B). This explains the role of *VAMP2* variants in impaired neurodevelopment [6] and *AGRN* mutations in causing CMS [7,8]. Our patient has a missense mutation in the *VAMP2* gene on the p arm of chromosome 17, which is spanned by previously studied variants, as discussed below (Figure 2) [1]. Our patient's missense mutation, NM_014232: c.227 A>C (p.Gln76Pro), is in exon 3 of the *VAMP2* gene.

Salpietro V et al. reported that two patients with variants, missense mutations in *VAMP2*, patient (p.Ser75Pro mutation) is a three-year-old female with a point mutation in *VAMP2* (NM_014232: c.223 T>C [p.Ser75Pro]) and patient (p.Phe77Ser mutation) is a 13-year-old male with a point mutation in *VAMP2* (NM_014232: c.230 T>C [p.Phe77Ser]) and also in exon 3 [1]. These earlier *VAMP2* mutations and disease manifestations exhibit similarities with our patient's symptoms. The patient with p.Ser75Pro mutation also presents abnormal EEG of poorly organized high-voltage delta activity, sharp wave slow-wave complexes, and central visual defects [1]. Moreover, the patient with p.Phe77Ser mutation also presents with disorganized EEG paroxysms and central visual defects similar to ours [1]. Salpietro V et al. further analyzed the molecular structure of the mutation variants. Patient variant p.Ser75Pro resulted in the loss of two hydrogen bonds, one interchain between Ser75 of *VAMP2* and Tyr243 of Syntaxin 1A (STX1A) and one intrachain between Ser75 and Gln7 [1]. Moreover, patient variant p.Phe77Ser presents a hydrophilic residue mutation in an otherwise hydrophobic region. In comparison, our patient has a p.Gln76Pro mutation where a polar amino acid, glutamine, is replaced with another polar amino acid, proline. Proline weakens α-helices because of its asymmetrical geometry, where nitrogen of the amide group bonds with its R-group, producing steric hindrance. Further, the absence of hydrogen on proline's nitrogen prevents it from partaking in hydrogen bonding [10]. Due to the proximity of the location of the amino acids, the variant of the mutation, and the bond properties between the amino acids, this could indicate a possible lead to the similarities seen in our patients with p.Gln76Pro and p.Phe77Ser mutations, as seen in the study by Salpietro V et al. [1]. It is important to note that all three of these modifications have happened adjacent to one another, and the disease phenotypes between our patient and Salpietro V et al. have been similar [1]. There clearly indicates that this region is a conserved region and part of the active protein domain. These missense mutations can lead to changes in the amino acid in the primary protein sequence, which can significantly alter the proteins' nature, folding, and function, possibly resulting in improper formation of the SNARE complex in the postsynaptic membrane [9]. Wang C et al. described the membrane organization of the *VAMP2* SNARE motif and the organizational change of *VAMP2* upon changing the intracellular lipid environment [9]. Their study suggested that the glutamine at position 76 is in the SNARE motif, which is a crucial motif in the *VAMP2* proteins. Their studies further showed that residues 35-78 of the SNARE motif are less adaptable and well-ordered [9]. Truncation of the C-terminal half of the SNARE motif and the juxta-membrane domain (VAMP2(60-96)) eliminated the membrane relationship of *VAMP2*, which indicates that in addition to the juxta-membrane domain, the SNARE motif is also essential for the membrane association of *VAMP2*.

Our patient also has a mutation in *AGRN* (NM_198576: c.2908 C>G [p.Gln970Glu]), which is also a missense mutation. Glutamine is an amino acid that is neutral at physiological pH, whereas glutamate is acidic at physiological pH [11]. Glutamate permits tighter packing in alpha-helices [12]. Ohkawara B et al. studied four CMS patients with variants in *AGRN *[13]. Among these patients, patient 4 was found to have generalized hypotonia with both proximal and distal limb muscle weakness [13]. At the same time, the other patients presented with varying symptoms, including a waddling gait, tracheostomy for respiratory support, and scoliosis. The mutation in patient 4 involved NM_198576: c.5023 G>A (p.Gly1675Ser). In order to see the effect of patient 4's missense mutation in *AGRN* on AChR clustering, agrin and WT fragments were added to a conditioned culture medium and then added to C2C12 myotubes, and it was concluded that the mutated agrin fragments resulted in decreased areas of AChR clustering [13]. From genome data mining analysis via gnomAD, we discovered that a mutation in front of p.Pro968Pro and after c.2911+4_2911+5del, the one we observed in our patient in *AGRN* (NM_198576: c.2908 C>G [p.Gln970Glu]) are in the splice region [14,15]. This may be much more deleterious and may result in dysfunctional proteins, leading to the improper signaling of the agrin-Lrp4-MuSK pathway. Although the purpose of this protein's domain is not clearly determined, they are stated to contain glycosylated sites [16] and are essential for binding to neural cell adhesion molecule 1 [17]. In a study conducted by Karakaya M et al., a 17-month-old male was studied who presented with signs of neck extensor muscle weakness and mild limb-girdle weakness [5]. By 30 months of age, the patient presented with bilateral ptosis and restrictive eye movements that got worse throughout the day [5]. This patient presents features similar to our patient, including poor head control and problems with eye movements. After performing whole exome sequencing on this male, Karayaka M et al. found a homozygous nonconservative missense mutation in exon 29 of the *AGRN* gene (g.chr1:985853 G>A, c.5023 G>A, p.Gly1675Ser) [5]. Missense mutations in the *AGRN* gene present with variable features of CMS, as can be seen by patients in earlier studies [4,5]. 

The docking mechanism of synaptic vesicles plays a significant role in the proper signal pathway of neurons. Simmons RL et al. have looked into 4-aminopyridine (4-AP) as a potential treatment strategy for missense and nonsense variants in the *VAMP2* gene of synaptic vesicle docking [2]. Variants in *VAMP2* are associated with impaired action potential triggered by aberrant neurotransmitter exocytosis and endocytosis [2]. Simmons RL et al. hypothesize that treatment with 4-AP could prolong the duration of the action potential leading to an increased release of synaptic vesicles [2]. 4-AP has been shown to work by inhibiting K+ channels, leading to increased Ca2+ availability [2]. Manipulating the mechanism of the Ca2+ channels can provide a longer time period for exocytosis of the synaptic vesicle and trigger neurotransmitter release [1,18,19]. Treatment options found beneficial to patients with missense mutations in *AGRN* include Salbutamol [5] and should be considered in the future for patients presenting with mutations in the *AGRN* pathway. Salbutamol is a beta-2 adrenergic receptor agonist and has been found to improve muscle strength in patients suffering from CMS with AChR deficiency [20].

## Conclusions

In summary, GDD is a broad spectrum of genetic disorders presenting different genetic variants and clinical symptoms in the early developmental stages of life. GDD can manifest in various ways depending on the segment of the affected gene. The mechanism underlying the genetic mutations remains obscure. While lab results can aid in ruling out known diseases, it is not sufficient for discovering unknown GDD conditions. The protein genetic variant of p.Gln76Pro associated with *VAMP2* and p.Gln970Glu associated with *AGRN* are novel because these variants have not been found in combination in other studies. Uncovering the molecular structure of proteins in the different neuronal signaling pathways could aid future research in understanding the relationship between genes, their location, and the mechanisms of disease. This report illustrates the complex mechanisms of action that could contribute to the pathology of GDD.
